# Friction and Wear Performance Evaluation of Bio-Lubricants and DLC Coatings on Cam/Tappet Interface of Internal Combustion Engines

**DOI:** 10.3390/ma14237206

**Published:** 2021-11-26

**Authors:** Rehan Zahid, Muhammad Usman Bhutta, Riaz Ahmad Mufti, Muhammad Usman Abdullah, Haji Hassan Masjuki, Mahendra Varman, Muhammad Abul Kalam, Mian Ashfaq Ali, Jawad Aslam, Khalid Akhtar

**Affiliations:** 1 School of Mechanical & Manufacturing Engineering (SMME), Campus H-12, National University of Sciences & Technology (NUST), Islamabad 44000, Pakistan; rehanzahid@smme.nust.edu.pk (R.Z.); riazmufti@smme.nust.edu.pk (R.A.M.); engr.usmanabdullah@gmail.com (M.U.A.); mian.ashfaq@smme.nust.edu.pk (M.A.A.); jawadaslam@smme.nust.edu.pk (J.A.); drkhalidakhtar@smme.nust.edu.pk (K.A.); 2 Department of Mechanical Engineering, Gombak Campus, International Islamic University Malaysia, P.O. Box 10, Kuala Lumpur 50728, Malaysia; masjuki@iium.edu.my; 3 Department of Mechanical Engineering, Office of the Faculty of Engineering, University of Malaya, Kuala Lumpur 50603, Malaysia; mahendra@um.edu.my (M.V.); kalam@um.edu.my (M.A.K.)

**Keywords:** diamond-like carbon (DLC) coatings, bio-lubricants, lubricant additives, cam/tappet interface, scanning electron microscopy (SEM), energy dispersive X-ray spectroscopy (EDS)

## Abstract

The environmental concerns associated with artificially formulated engine oils have forced a shift towards bio-based lubricants. The deposition of hard coatings on engine components and migrating to environmentally friendly green lubricants can help in this regard. Chemically modified forms of vegetable oils, with better low-temperature characteristics and enhanced thermo-oxidative stability, are suitable substitutes to conventional lubricant base oils. The research presented in this manuscript was undertaken to experimentally investigate the wear and friction performance of a possible future generation of an environmentally friendly bio-based lubricant as a potential replacement for conventional engine lubricants. In order to quantify the tribological benefits which can be gained by the deposition of DLC coatings, (an (a-C:H) hydrogenated DLC coating and an (a-C:H:W) tungsten-doped DLC coating) were applied on the cam/tappet interface of a direct acting valve train assembly of an internal combustion engine. The tribological correlation between DLC-coated engine components, lubricant base oils and lubricant additives have been thoroughly investigated in this study using actual engine operating conditions. Two additive-free base oils (polyalphaolefines (PAO) and chemically-modified palm oil (TMP)) and two multi-additive-containing lubricants were used in this investigation. Real-time drive torque was measured to determine the friction force, detailed post-test analysis was performed, which involved the use of a specialized jig to measure camlobe wear. An optical profilometer was used to measure the wear on the tappet, high-resolution scanning electron microscopy was employed to study the wear mechanism and energy-dispersive X-ray spectroscopy was performed on the tested samples to qualitatively access the degradation of the coating. When using additive-free TMP, a low friction coefficient was observed for the cam/tappet interface. The presence of additives further improved the friction characteristics of TMP, resulting in reduced average friction torque values. A tremendous enhancement in wear performance was recorded with a-C:H-coated parts and the coating was able to withstand the test conditions with little or no delamination.

## 1. Introduction

Automotive manufacturers, especially those using internal combustion (IC) engines as a power source, are under immense pressure from environmental agencies and government legislators. The main reason behind these strict legislations for automotive OEMs is the extremely high carbon footprints associated with the operation of IC-engine-based vehicles. Electric vehicle manufacturers are exploiting the situation and rapidly capturing the market share of conventional car manufacturers. The only way for IC-engine-based vehicles to remain relevant in the automotive industry is to reduce the associated energy losses and environmental issues by working on various facets simultaneously.

Losses due to friction and associated wear can be reduced to an appreciable extent by applying surface-protective coatings and surface treatments on interacting machine parts and components [1,2,3,4]. Diamond-like carbon (DLC) coatings have been developed as a prominent surface-protective solution for widespread applications because of their outstanding mechanical, friction, wear, and chemical properties [5,6,7,8,9]. DLC coatings are known to be chemically inert [10,11], and the doping of DLC coatings with metals such as tungsten can not only enhance their surface energies but can also increase their ability to interact with lubricants to develop surface-protective films. Other advantages associated with doped-DLC coatings include improved wear resistance, good adhesion with the substrate, enhanced electrical conductivity, and decreased compressive internal stresses during deposition [12,13,14,15]. Lubricants, used in automotive engines for the purpose of reducing the friction and wear of components, are mostly derived from non-biodegradable and non-renewable sources, such as petroleum. Due to ever-increasing environmental issues, health concerns, and the scarcity of raw materials require for the production of conventional lubricants, there is a need to look for alternate sources of lubricants which are not only environmentally sustainable but also renewable and biodegradable [16]. According to the published studies, vegetable oils have comparable values of key tribological performance parameters (viscosity index, lubricity, flash point, and pour point) to those of conventional base oils [17,18,19,20]. However, inherent low-temperature characteristics and oxidation instability are a few of the undesirable inherent characteristics of vegetable oils. These shortcomings are raised by the presence of unsaturated bonds in the structure of vegetable oils, leading to high reactivity with atmospheric oxygen, resulting in oxidative degradation [21].

In the past, the potential of vegetable oils as lubricant base oils has been evaluated by a number of researchers [22,23,24,25]. The main objective of those studies was to partially/completely replace the commercially available lubricants with bio-lubricants. In most of the abovementioned studies, the compatibility of commercially available additives with rapeseed, sunflower, and soybean oil-based bio-lubricants have been evaluated using tribometers with uncoated metallic tribopairs. Very few researchers have evaluated the tribological performance of palm oil, either as an additive in commercially available lubricants or in the additive-free form in combination with DLC coatings using various tribometers [26,27,28,29]. At the time of submitting this paper, no experimental study has been previously reported/published that evaluates the tribological performance of palm oil with DLC-coated components in an actual engine. In order to address this research gap, chemically modified palm oil (TMP) was used in combination with DLC-coated engine components. The goal of this investigation was to explore the tribological compatibility and synergistic correlation of various types of DLC coatings with TMP and conventional lubricant additives in order to develop a sustainable and eco-friendly solution. In this experimental study, the effects of DLC coatings and TMP on the tribological performance parameters of the tappet/cam interface of a valve train assembly were investigated using an OM646LA Mercedes Benz diesel engine cylinder head with additive-free base oils and multi-additive-containing lubricants. Moreover, material characterization techniques, namely, field emission scanning electron microscopy (FESEM) and energy dispersive X-ray spectroscopy (EDS), were deployed on the post-test surfaces to unveil the underlying mechanisms governing the changes in their friction and wear behavior.

## 2. Materials and Methods

### 2.1. Formulation of Lubricants

**Figure 1:**
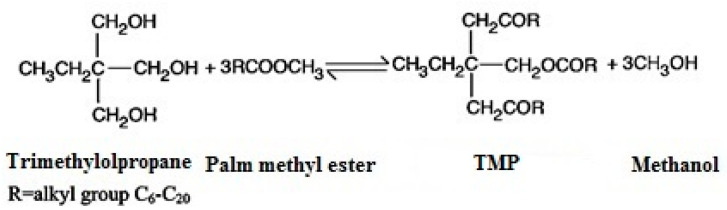
Chemical equation governing TMP synthesis.

The tribological performance of additive-free and multi-additive-containing lubricants was analyzed in this research. TMP was used as bio-lubricant and its tribological performance was compared with that of polyalphaolefins (PAOs), which are most widely used as conventional lubricant base oils. The thermo-oxidation stability and inherent low-temperature properties of palm oil were enhanced by applying a transesterification process which involved the chemical reaction of trimethylolpropane with palm methyl ester to form chemically modified palm oil (Figure 1). The procedure for producing TMP was described in more detail in our previous work [30]. Cam/tappet interfaces mostly operate under boundary lubrication regimes in which additives can play a vital role by forming tribochemical films on the interacting surfaces. Therefore, ZDDP, GMO, and MoDTC, which are the most widely investigated conventional lubricant additives, were used in combination with the base oils mentioned above to further improve their inherent friction performance and wear prevention characteristics. The additives were imported from Adeka, Japan, and PAO was acquired from Ineos, USA. Each formulated lubrication was developed using 1 wt.% of each additive with 97 wt.% base oil. The physicochemical properties and formulation details of the formulated and additive-free lubricants has been presented in Table 1. Kinematic viscosities and densities of all the lubricants considered in this study were measured using an Anton Paar SVM 3000 Stabinger viscometer according to the ASTM D7042-2012 standards, whereas VI was calculated through ASTM D2270 by measuring the values of kinematic viscosities at 40 and 100 °C.

### 2.2. DLC Coating Specifications

In this study, a-C:H and a-C:H:W coatings were chosen for analysis on the basis of a literature review and tribotesting [30,31,32,33,34]. The abovementioned coatings belong to the non-doped and doped categories of DLC coatings, respectively, and have been widely investigated in the past in combination with lubricants formulated using conventional lubricant additives and base oils. All of these coatings are commercially available and belong to the Balinit series of Oerlikon Balzers. The Balinit series of DLC coatings are specially designed for industrial applications where low friction coefficients and the wear protection of interacting components are of prime interest. A few of the important properties of a-C:H and a-C:H:W coatings are mentioned in Table 2.

### 2.3. Experimental Setup

**Figure 2:**
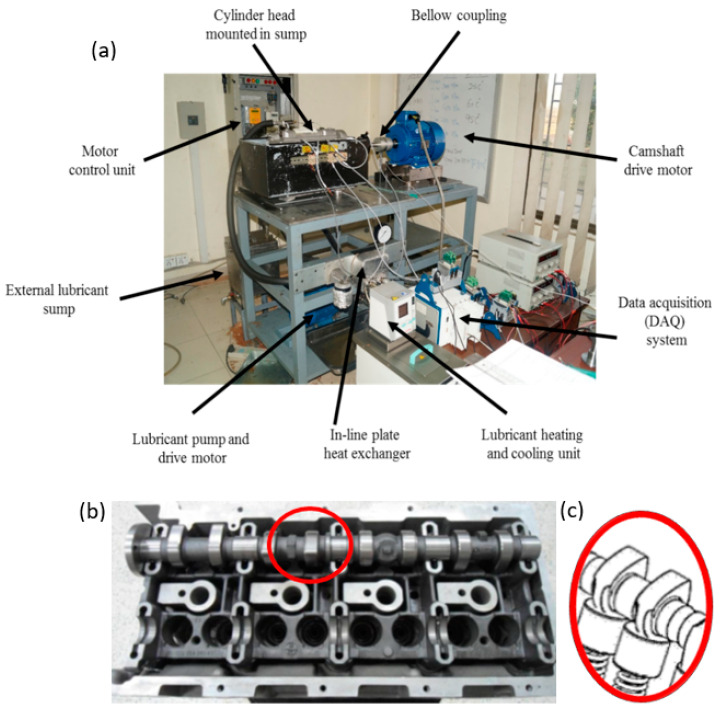
(**a**) Direct acting valve train test rig; (**b**) Mercedes Benz OM646LA cylinder head; (**c**) cam/tappet interface.

An OM646LA Mercedes-Benz diesel engine cylinder head was mounted on a direct acting engine valve train test rig, as shown in Figure 2, and was used to conduct engine tests under motored conditions. OM646LA is a four-cylinder engine with twin cam, eight intake and eight exhaust valves. The important specifications of the OM646LA engine are listed in Table 3. A feedback-controlled variable-speed induction motor was coupled with the exhaust camshaft of the engine using bellow coupling, capable of transmitting back-lash-free torque and compensating for shaft misalignments (radial, axial, and angular). A controller, data acquisition system (DAQ) and Laboratory Virtual Instrument Engineering Workbench (LabVIEW)-based customized software was used to control and monitor the speed of the motor and conduct the engine tests at various camshaft speeds. Another motor was used to run the lubricant pump. The DAQ system was employed to control the pressure and flow rate of the lubricant. The engine was assembled in an oil sump, made from sheet metal, which was then bolted onto the valve train test rig. It was also used to contain the lubricant coming out of the engine, from which it was transferred to the external sump, which was basically an insulated double-walled container capable of maintaining the temperature of the oil via gravitational flow. The external sump was used as lubricant reservoir from which lubricant was pumped into the engine. Lubricant was heated at different temperatures and its temperature was maintained using a lubricant heating and cooling unit. Heating of the lubricant was carried out indirectly. Initially, heat transfer oil was heated by the lubricant heating/cooling unit and then heat was transferred to the lubricant using an in-line plate heat exchanger. The temperature of the lubricant was monitored using a thermocouple connected to the lubricant inlet of the engine. To minimize heat losses, lubricant pipes were insulated. An analog pressure gauge was used to monitor the pressure of the lubricant entering the engine.

In addition, a pressure transducer (piezo-resistive) was deployed to convert pressure into voltage. In this way, the pressure of the lubricant entering into the engine was monitored and controlled using LabVIEW-based computer software. A feedback-looped proportional, integral, and differential (PID) controller was used to monitor, record, and control the pressure and temperature of the lubricant throughout the test run. The complete test rig, along with Mercedes Benz OM646LA cylinder head, is shown in Figure 2.

### 2.4. Testing Procedure

The friction and wear results of the uncoated cam/uncoated tappet (U-U) interface, published in our previous study [32], were compared with those of a-C:H-coated cam/a-C:H-coated tappet (a-C:H-a-C:H) and a-C:H:W-coated cam/a-C:H:W-coated tappet (a-C:H:W-a-C:H:W) interfaces. A comprehensive testing matrix, as shown in Table 4, was devised to simulate real engine operating conditions by conducting tests at three different speeds of camshaft (400 RPM, 800 RPM, and 1200 RPM) and two different lubricant temperatures (40 and 90 °C). Each lubricant was investigated using new engine components (tappets and camlobes). Before each test, the lubricant was heated to the testing temperature and was circulated for two hours in the cylinder head. During each test, the pressure of the lubricant was kept at 2 bars. The friction and wear performance of tappet/cam interface in combination with different lubricants was first investigated at a 1200-RPM camshaft speed with a lubricant temperature of 40 °C. The values of the tribological performance parameter, i.e., friction torque, were measured after running the system for 30 min using the NI DAQ system and LabVIEW software. Friction torque readings were also taken at camshaft speeds of 800 RPM and 400 RPM. After conducting the engine tests at 40 °C, the tested parts were taken out of the engine head for detailed post-experimental analysis and new components were installed to perform the test at 90 °C. The same procedure was adopted to perform the tests at 90 °C. The lubricant temperature was increased to 90 °C and circulated in the engine cylinder head for two hours, which ensured that the temperature of engine components was maintained at 90 °C. Friction torque readings were taken at camshaft speeds of 1200, 800, and 400 RPM by repeating the above-mentioned procedure at the elevated lubricant temperature of 90 °C. Each test was performed at least twice to ensure repeatability.

### 2.5. Characterization of Worn Surfaces

Frictional torque at the tappet/cam interface was monitored using a strain-gauge-based torque transducer. Only the exhaust camshaft with one cam/tappet pair was used for the measurement of tribological performance parameters. The wear of the tappets was measured using a Nanovea PS50 profilometer, whereas the wear of the camlobe nose was calculated using a Solartron VG/20/S AMETEK (LVDT) Linear Variable Differential Transformer in combination with a specially designed jig. Details of the camshaft friction torque measurements using the torque transducer and the wear measurement procedure and technique can be found in our previous experimental studies [32,35].

A range of surface characterization and analysis techniques were deployed to explore the underlying wear and friction mechanisms accountable for a particular tribological behavior. Before conducting the surface analysis, washing of the components with cyclohexane was carried out and after that they were placed in a vacuumed desiccator. After the completion of the engine tests, tappets’ surfaces were analyzed with Zeiss UltraPlus FESEM to determine the predominant wear mechanisms. Due to the tribochemical interaction between the tappet surface and lubricant additives, additive-derived tribofilms are generally produced and deposited on the interacting surfaces. EDS, in combination with FESEM, was used to determine the chemical composition of additive-derived tribofilms deposited on the tappet surface. A Surftest SJ-210 mechanical stylus profilometer was used to investigate the profiles of the tappets’ surfaces after the engine tests and the surface profile data were utilized to calculate the values of average surface roughness (R_a_) of the tappets.

## 3. Results and Discussion

### 3.1. Friction Analysis of the a-C:H-a-C:H Interface

**Figure 3:**
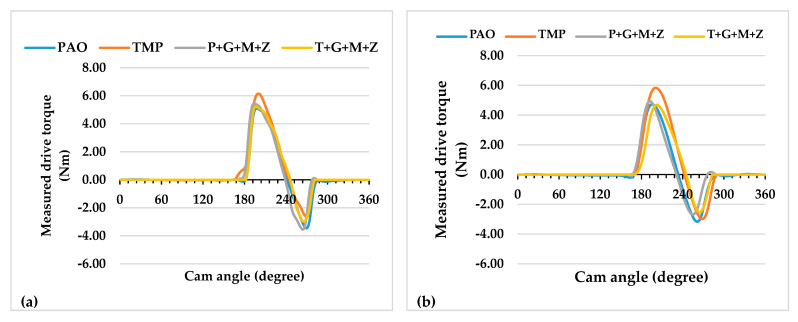

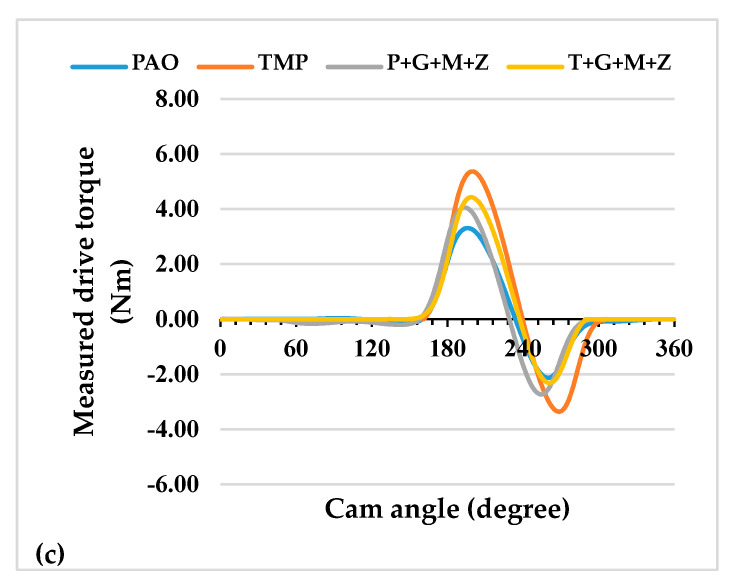
Instantaneous exhaust camshaft drive torque with a-C:H-coated tappets and a-C:H-coated camlobes at a lubricant temperature of 40 °C and camshaft speeds of (**a**) 400 RPM, (**b**) 800 RPM, and (**c**) 1200 RPM.

**Figure 4:**
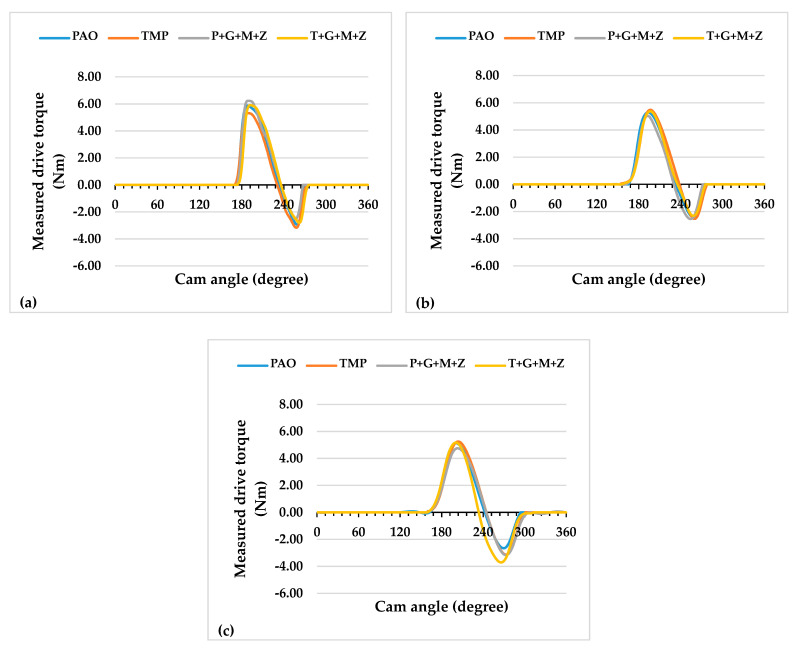
Instantaneous exhaust camshaft drive torque with a-C:H-coated tappets and a-C:H-coated camlobes at a lubricant temperature of 90 °C and camshaft speeds of (**a**) 400 RPM, (**b**) 800 RPM, and (**c**) 1200 RPM.

**Figure 5:**
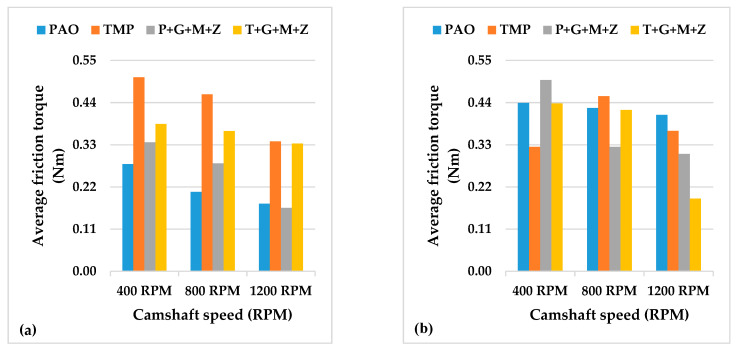
Average friction torque of exhaust camshaft with a-C:H-coated tappets and a-C:H-coated camlobes at camshaft speeds of 400 RPM, 800 RPM, and 1200 RPM and lubricant temperatures of (**a**) 40 °C and (**b**) 90 °C.

The instantaneous drive torque of the a-C:H-a-C:H interface at lubricant temperatures of 40 and 90 °C are represented in Figure 3 and Figure 4, respectively, whereas the average frictional torque of the exhaust camshaft is shown in Figure 5.

Additive-free TMP resulted in a lower friction coefficient of the interface in comparison with additive-free PAO when the engine test was conducted at 90 °C and 400 RPM (Figure 5). This lower friction coefficient was due to the polar components present in the structure of TMP. Similar behavior was also seen in our previous study with an uncoated cam/tappet interface (U-U) [32]. Upon increasing the camshaft speed, it was observed that values of the friction torque and instantaneous drive torque of the above-mentioned interface were either reduced or remained constant regardless of the lubricant formation (Figure 5). An exception to the above-mentioned finding was noted by increasing the camshaft speed from 400 RPM to 800 RPM in the presence of additive-free TMP. This abnormality in friction behavior can be associated with the extremely low average friction torque of the interface in the presence of additive-free TMP compared to other lubricants at 400 RPM. A possible justification for this behavior can be the graphitization of a-C:H-coated components in the presence of TMP at low engine speeds. A decrease in the friction coefficient by increasing the camshaft speed was also observed when the test was performed at a lubricant temperature of 40 °C. This enhanced friction performance of the a-C:H-a-C:H interface at 800 RPM and 1200 RPM can be attributed to a reduction in the cam load at the nose and an increase in the lubricant entrainment velocity at high camshaft speeds, which resulted in beneficial lubrication conditions [36]. Valve spring compression greatly affects cam load at low camshaft speeds, whereas inertia becomes more effective at high camshaft speeds [36]. The rate of deceleration of the tappet is proportional to the speed of the camshaft due to the tappet’s inertia. As a result, the cam load decreases with an increase in the camshaft speed [37].

Although multi-additive-containing lubricants were able to reduce the friction torque when used in combination with an uncoated cam/tappet interface at a camshaft speed of 400 RPM and a lubricant temperature of 90 °C in our previous research, no such behavior was seen when a-C:H-coated cam/tappet interface was used [32]. Rather, an increase in the average friction torque was noted when P+G+M+Z and T+G+M+Z were used as lubricants instead of additive-free base oils. The inability of additives to tribochemically interact with a-C:H-coated components can be attributed to the chemically inert nature of DLC coatings. At higher camshaft speeds, a decrease in the average friction torque values of the interface in the presence of multi-additive-containing lubricants was observed. This improvement in friction torque performance can be attributed to enhanced lubrication of the interface at higher camshaft speeds; thus, lubricant additives are more likely to tribochemically interact with mating components, resulting in the production of tribofilms. The mechanisms of tribofilm formation and their effect on the tribological performance of contact surfaces will be discussed in following sections.

A significant decrease in the average friction torque values was seen when the temperature of the lubricant was decreased from 90 °C to 40 °C with most of the lubricant formulations (Figure 5). This improvement in friction performance is due to the high viscosity of the lubricant at 40 °C compared to 90 °C, wherein a thick lubricant film is present between the interacting components, resulting in an elastohydrodynamic lubrication regime with a reduced chance of direct metal-to-metal contact compared to the pure boundary lubrication regime. Exceptions to this behavior were noted with TMP at 400 RPM and T+G+M+Z at 1200 RPM. The high friction torque of the interface in combination with TMP at 90 °C and 400 RPM can be attributed to graphitization. It has been stated by researchers that graphitization is a temperature-dependent phenomenon and the chances of its occurrence decrease at lower contact temperatures [38].

### 3.2. Wear Analysis of the a-C:H-a-C:H Interface

**Figure 6:**
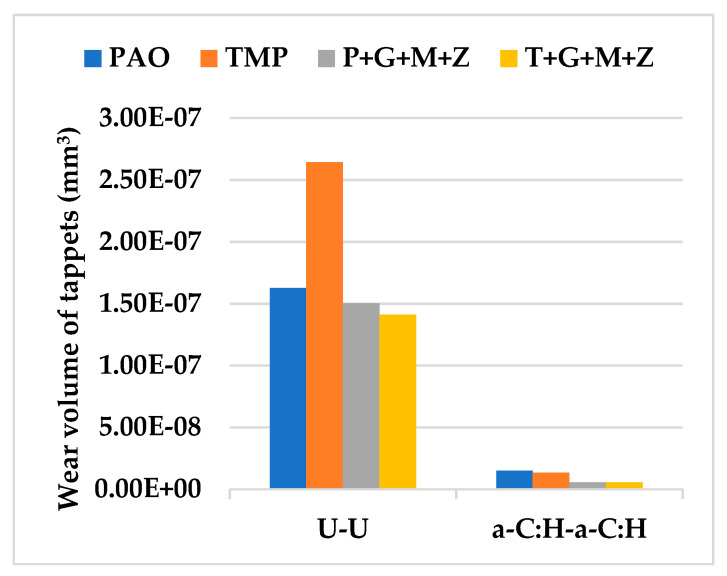
Wear volume of uncoated and a-C:H-coated tappets after cylinder head testing at various conditions in the presence of TMP-based and PAO-based lubricants.

**Figure 7:**
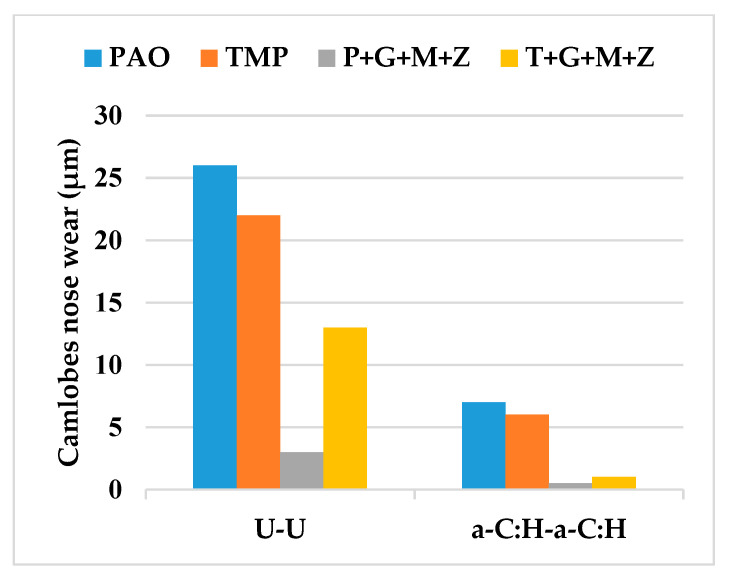
Nose wear of uncoated and a-C:H-coated camlobes after cylinder head testing at various testing conditions with TMP-based and PAO-based lubricants.

The wear volume of both uncoated and a-C:H-coated tappets after cylinder head testing using PAO-based and TMP-based lubricants at various temperatures and camshaft speeds are represented in Figure 6, whereas Figure 7 shows the nose wear of uncoated and a-C:H-coated camlobes.

**Figure 8:**
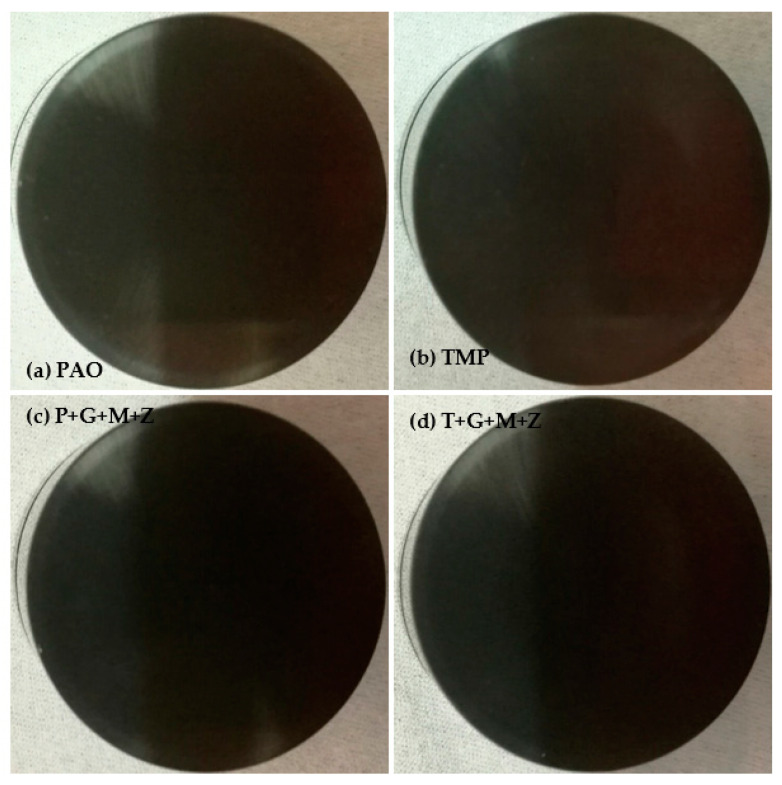
Optical images of a-C:H-coated tappets after cylinder head testing in combination with a-C:H-coated camlobes under various conditions with TMP-based and PAO-based lubricants.

**Figure 9:**
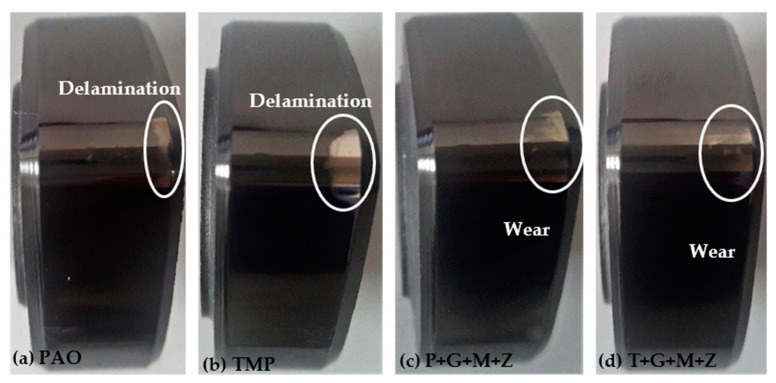
Optical images of a-C:H-coated camlobes after cylinder head testing in combination with a-C:H-coated tappets under various conditions with TMP-based and PAO-based lubricants.

After cylinder head testing, optical images of a-C:H-coated tappets and a-C:H-coated camlobes were captured using a 12-megapixel camera and are represented in Figure 8 and Figure 9, respectively. It was observed that the wear volume values of coated components in the presence of TMP were less than those of PAO and the wear performance of tappets improved further when using multi-additive-containing lubricants. Similar behavior was also noted in the nose wear of a-C:H-coated camlobes. No signs of any peeling off or delamination of the coating from the tappets was observed in the optical images, irrespective of the lubricant formulation (Figure 8). Although most of the coating also remained intact on the camlobe surface after cylinder head testing, some delamination/wear was seen on the right edges of the cam nose, particularly with the additive-free lubricants (Figure 9).

### 3.3. SEM/EDS Analysis of the a-C:H-a-C:H Interface

**Figure 10:**
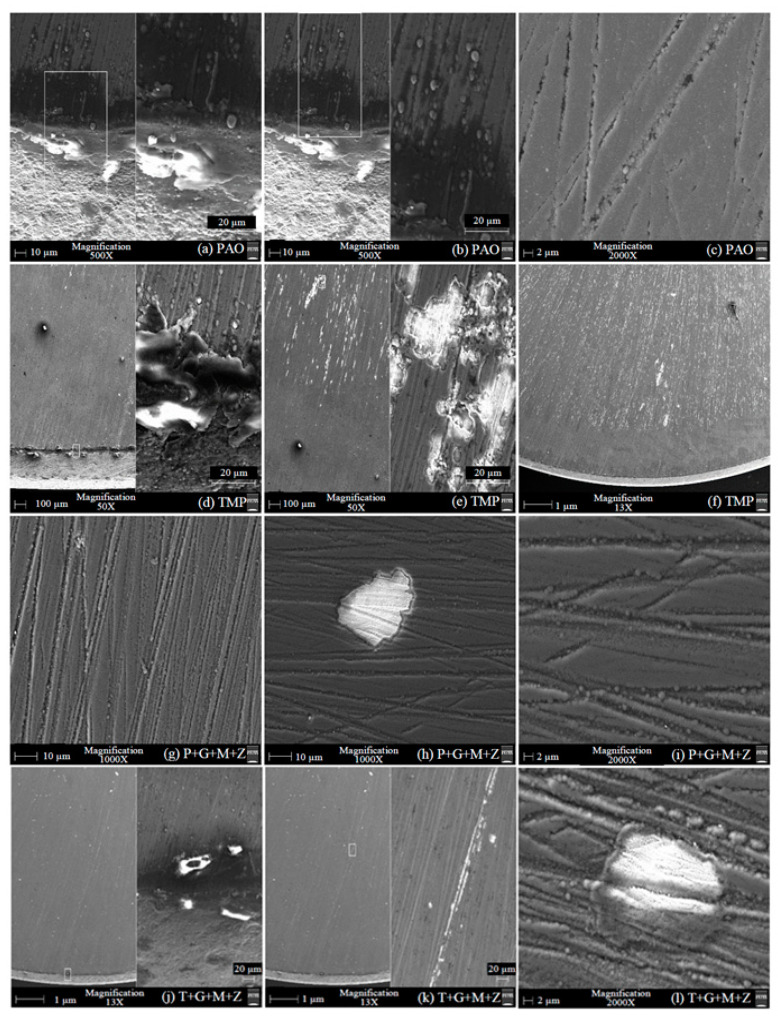
SEM micrographs of a-C:H-coated tappets after engine testing under various conditions in combination with a-C:H-coated camlobes with TMP-based and PAO-based lubricants.

After cylinder testing, micrographs of a-C:H-coated tappets were obtained with the help of SEM and are represented in Figure 10. Some deposited elements on tappets were also seen after the test and the atomic percentages of those elements are given in Table 5. In these micrographs, abrasive wear can be seen as the predominant wear mechanism, resulting in scratch lines on the top tappet surface due to the sliding of the camlobes. The coating was slightly worn-out when TMP-based lubricants were used, especially additive-free TMP, resulting in the exposure of the CrN interlayer and the ferrous substrate, but no sign of severe coating delamination was seen in any of these micrographs. When using additive-free PAO, the a-C:H coatings on the tappet surface maintained their structural integrity and sustained the test conditions with few scratch marks and little wear debris (Figure 10c). Contrary to this, the top DLC layer was removed from the tappet surface, resulting in an exposed CrN interlayer and ferrous substrate in the presence of TMP (Figure 10e and Table 5). The white-colored dots, which can be seen in Figure 10e,f, were due to the exposed CrN interlayer, whereas the white-colored patches in Figure 10f represent the ferrous substrate. The low content of carbon and the presence of chromium, nitrogen, and ferrous in the EDS results confirmed the removal of the top DLC layer, resulting in the exposed CrN inlayer and ferrous substrate (Table 5). An increase in oxygen content was also seen in the case of additive-free TMP. This can be associated with the oxidation of the exposed ferrous substrate, resulting in the formation of FeO and Fe_2_O_3_. It was also observed that the wear resistance of the a-C:H-coated tappets improved upon the use of multi-additive-containing TMP instead of additive-free TMP. An exposed CrN interlayer was seen in a few spots, but the ferrous substrate was seen neither in the SEM micrographs nor in the EDS analysis. Similarly to the SEM micrographs of PAO, scratch lines and fine wear debris can be seen in the micrographs of P+G+M+Z. White-colored patches, which represent the exposed ferrous substrate due to the delamination of both the top a-C:H and CrN interlayer, can be seen in Figure 10h, but the overall coating strongly adhered to the substrate until the end of the cylinder head testing, as shown in Figure 10i. Since low concentrations of additive-derived elements were seen in the EDS analysis, the tribological improvement seen in the a-C:H-coated interface was mostly due to the hindrance in the occurrence of the graphitization phenomenon in addition to tribofilm formation.

### 3.4. Surface Roughness Analysis of a-C:H-Coated Tappet/a-C:H-Coated Camlobe (a-C:H-a-C:H)

**Figure 11:**
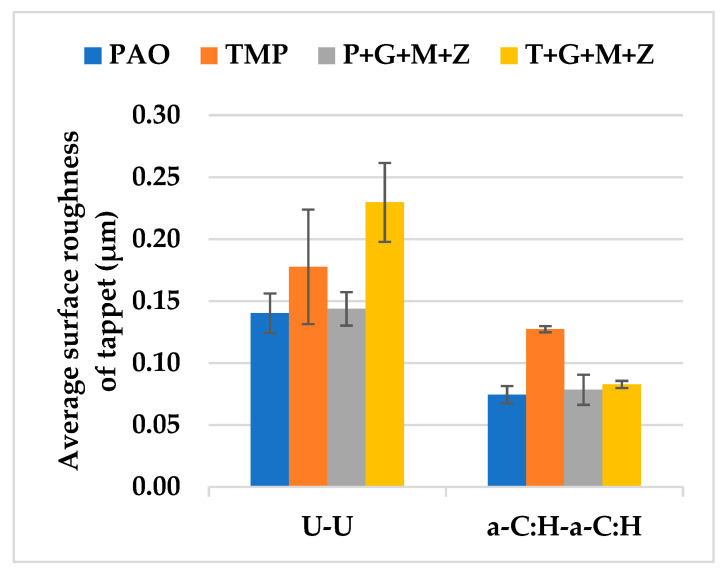
Average surface roughness of DLC-coated and uncoated tappets after cylinder head testing under various conditions in combination with uncoated and DLC-coated camlobes with TMP-based and PAO-based lubricants.

After the completion of cylinder tests under various conditions, the R_a_ values of a-C:H-coated and uncoated tappets with PAO-based and TMP-based lubricants were calculated and are represented in Figure 11.

Additive-free PAO was demonstrated to be more efficient in protecting the DLC layer from deterioration, compared to TMP. The highest R_a_ value of the a-C:H-coated tappet was observed when using TMP as a lubricant and this can be attributed to the removal of the top DLC coating and the exposure of the CrN interlayer and ferrous substrate. Another justification for this behavior could be the chemically inert nature of the a-C:H coating; thus, the polar components of TMP were not adsorbed on the contact area, resulting in lubricant slippage and metal-to-metal contact. Consequently, TMP showed inferior wear protection of the contact surfaces in comparison with PAO in most of the engine tests. The delamination and wearing-out of the DLC coating were replaced by polishing wear and a significant decrease in the R_a_ value, which were seen when multi-additive-containing TMP was used.

### 3.5. Friction Analysis of the a-C:H:W-a-C:H:W Interface

**Figure 12:**
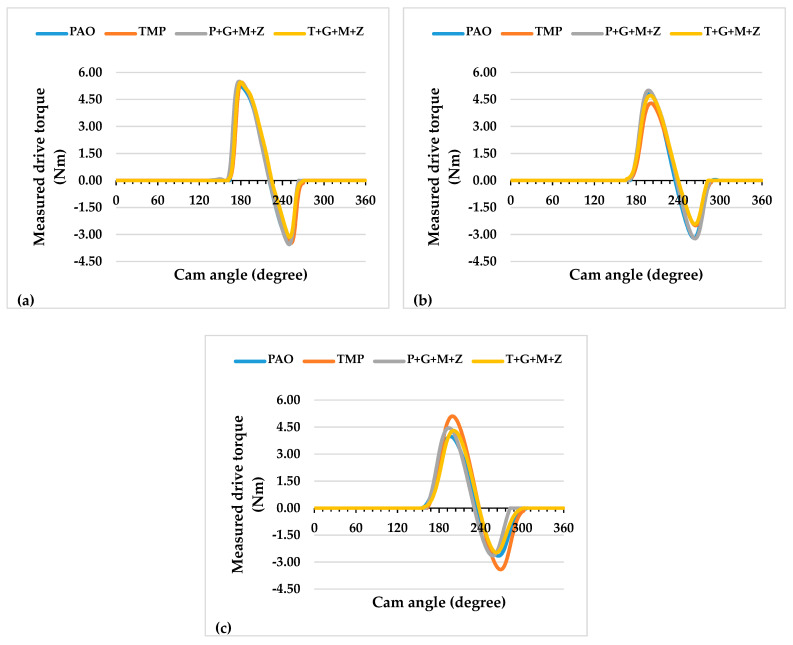
Instantaneous exhaust camshaft drive torque with a-C:H:W-coated tappets and a-C:H:W-coated camlobes at a lubricant temperature of 40 °C and camshaft speeds of (**a**) 400 RPM, (**b**) 800 RPM, and (**c**) 1200 RPM.

**Figure 13:**
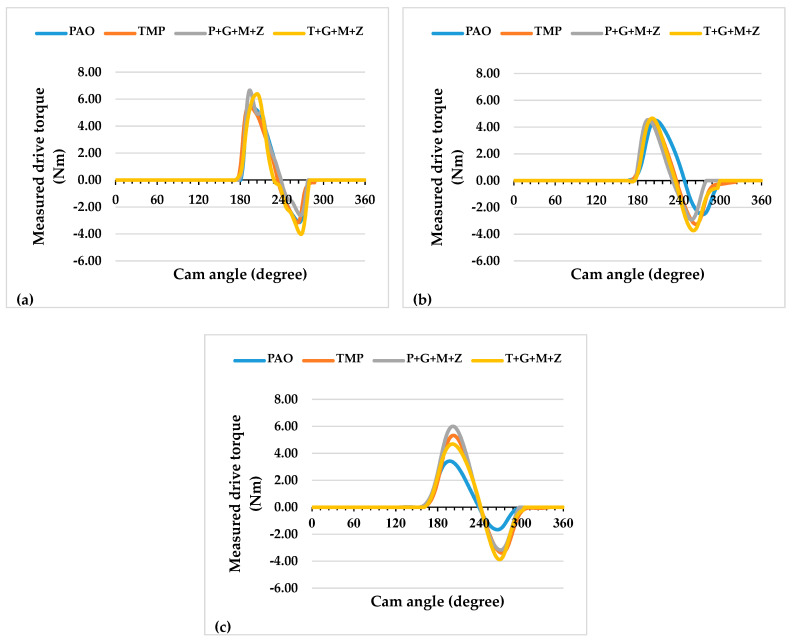
Instantaneous exhaust camshaft drive torque with a-C:H:W-coated tappets and a-C:H:W-coated camlobes at a lubricant temperature of 90 °C and camshaft speeds of (**a**) 400 RPM, (**b**) 800 RPM, and (**c**) 1200 RPM.

**Figure 14:**
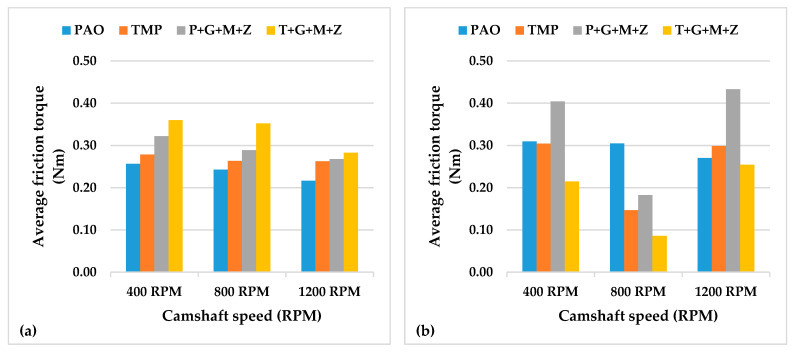
Average friction torque of exhaust camshafts with a-C:H:W-coated tappets and a-C:H:W-coated camlobes at camshaft speeds of 400 RPM, 800 RPM, and 1200 RPM and lubricant temperatures of (**a**) 40 °C and (**b**) 90 °C.

The instantaneous drive torque trends of camshafts at lubricant temperatures of 40 and 90 °C are represented in Figure 12 and Figure 13, respectively, whereas the values of the average friction torque of the tappet/cam interface, calculated using the instantaneous drive torque data, are shown in Figure 14.

A trivial decrease (3%) in the value of the average friction torque was noted upon the use of additive-free TMP in place of additive-free PAO at 90 °C and 400 RPM (Figure 14). A significant enhancement in the friction performance of the interface was observed with multi-additive-containing TMP, irrespective of camshaft speed, when tests were conducted at lubricant temperature of 90 °C. However, this behavior was not witnessed with P+G+M+Z (Figure 14). Upon increasing the camshaft speed from 400 RPM to 800 RPM, the friction performance of the interface improved to some extent, regardless of the lubricant formation (Figure 14). This trend in friction performance was also witnessed with the a-C:H–a-C:H interface. It was observed that when the speed was further raised to 1200 RPM, friction torque was raised again, almost to the same level as that of 400 RPM. This abnormality in the friction behavior of the a-C:H:W–a-C:H:W interface compared to the a-C:H-a-C:H interface will be discussed in the following sections.

When engine tests were conducted at a lubricant temperature of 40 °C, a decrease in the friction values of the interface was witnessed, irrespective of the camshaft speed and lubricant formulation (Figure 14). However, a few exceptions to this finding were seen at a camshaft speed of 800 RPM. Unlike the friction results obtained at 90 °C, a further decrease in the friction torque values was observed upon changing the camshaft speed from 800 RPM to 1200 RPM. The use of multi-additive-containing lubricants resulted in the deterioration of the friction performance of the interface. It is mentioned in the literature that additives have a threshold temperature and become ineffective below this temperature; therefore, a possible justification for this behavior could be the ineffectiveness of the additives at lower lubricant temperatures.

### 3.6. Wear Analysis of a-C:H:W-Coated Tappet/a-C:H:W-Coated Camlobe (a-C:H:W-a-C:H:W)

**Figure 15:**
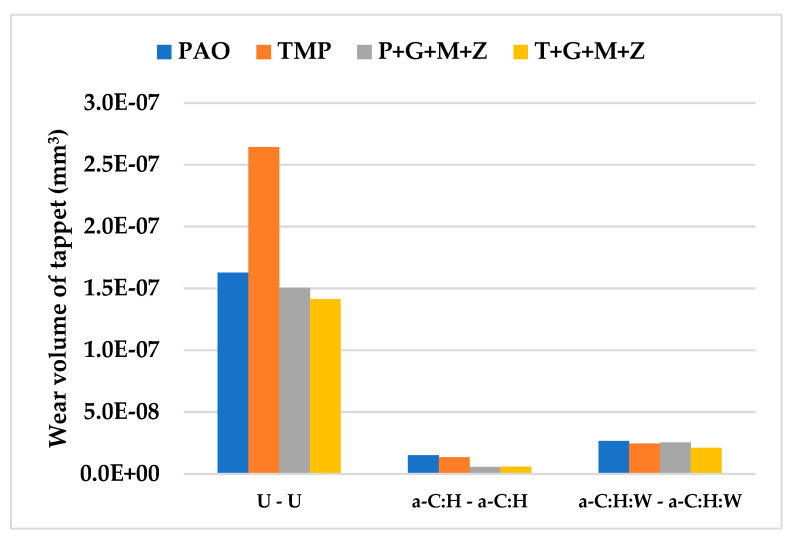
Wear volume of uncoated and DLC-coated tappets under various conditions with PAO-based and TMP-based lubricants.

**Figure 16:**
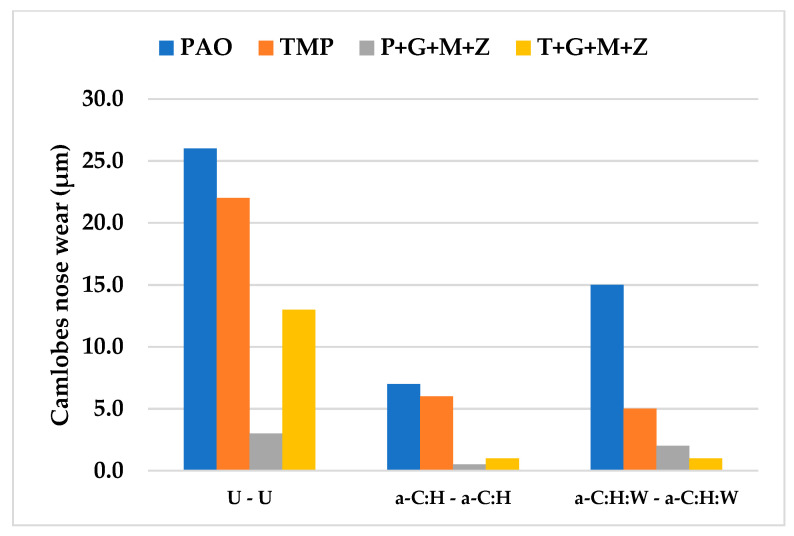
Nose wear of uncoated and DLC-coated camlobes under various conditions with PAO-based and TMP-based lubricants.

After conducting cylinder head tests at two different lubricant temperatures and three independent camshaft speeds in the presence of TMP-based and PAO-based lubricants separately, the wear volumes of the nose wear of uncoated/DLC-coated tappets and uncoated/DLC-coated camlobes were obtained, and these have been presented in Figure 15 and Figure 16, respectively.

**Figure 17:**
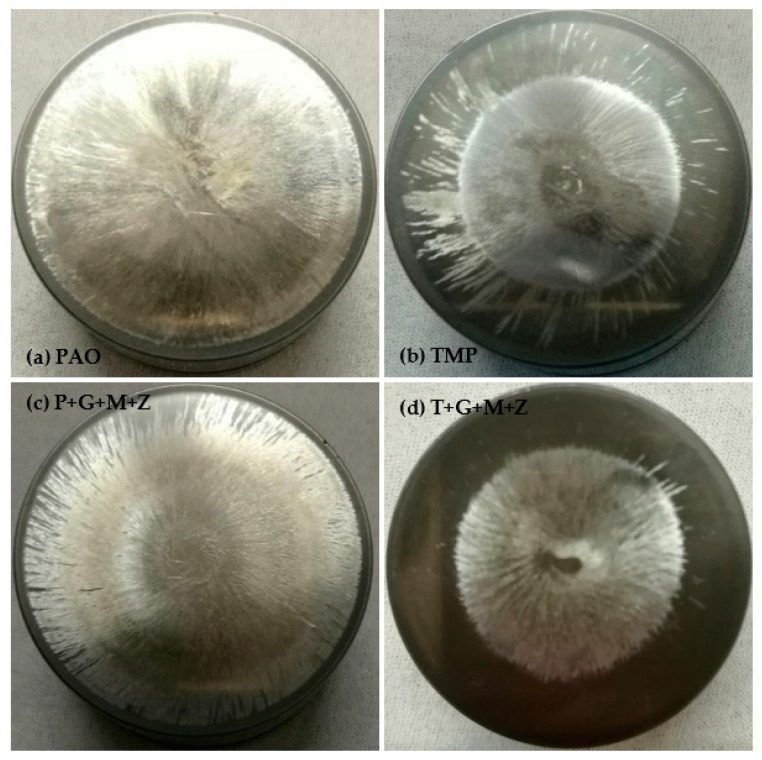
Optical images of a-C:H:W-coated tappets after cylinder head testing in combination with a-C:H:W-coated camlobes under various conditions in the presence of TMP-based and PAO-based lubricants.

**Figure 18:**
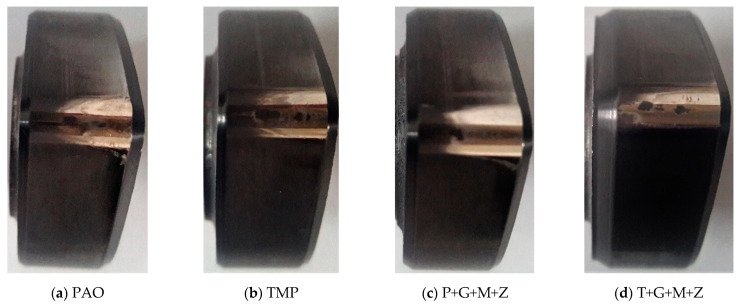
Optical images of a-C:H:W-coated camlobes after cylinder head testing in combination with a-C:H:W-coated tappets under various conditions in the presence of TMP-based and PAO-based lubricants.

Optical images of a-C:H:W-coated tappets and a-C:H:W-coated camlobes are shown in Figure 17 and Figure 18. It was observed that uncoated, a-C:H-coated, and a-C:H:W-coated tappets had the highest, lowest, and intermediate wear volume values, respectively (Figure 15). A similar trend was also noticed in the nose wear values of the camlobes (Figure 16). Additive-free TMP provided better surface protection to the a-C:H:W–a-C:H:W interface compared to additive-free PAO. The tungsten-doped DLC coating was completely peeled off from the tappets when PAO-based lubricants were used as lubricants (Figure 17). However, the coating remained intact on the outer periphery of the tappet’s top surface when TMP-based lubricants were used. A reduction in the wearing-out of a-C:H:W-coated camlobes was observed when multi-additive-containing lubricants, especially T+G+M+Z, were used (Figure 18). Similar behavior was also observed with U-U and a-C:H–a-C:H-coated interfaces. However, additives were not able to further improve the wear performance of a-C:H-coated tappets. When the nose wear values of a-C:H:W-coated camlobes were compared with the actual thickness of the DLC coating (3 µm), it was concluded that the coating was completely worn out when additive-free lubricants were used but in the presence of formulated lubricants, the wear resistance of DLC-coated camlobes improved to some extent. Since LVDT measurements were only made at one point om the camlobe nose due to design constraints, we can thus not be sure whether delamination of the coating occurred across the entire width of the camlobe nose or not when additive-free lubricants were used.

### 3.7. SEM/EDS Analysis of the a-C:H:W-a-C:H:W Interface

**Figure 19:**
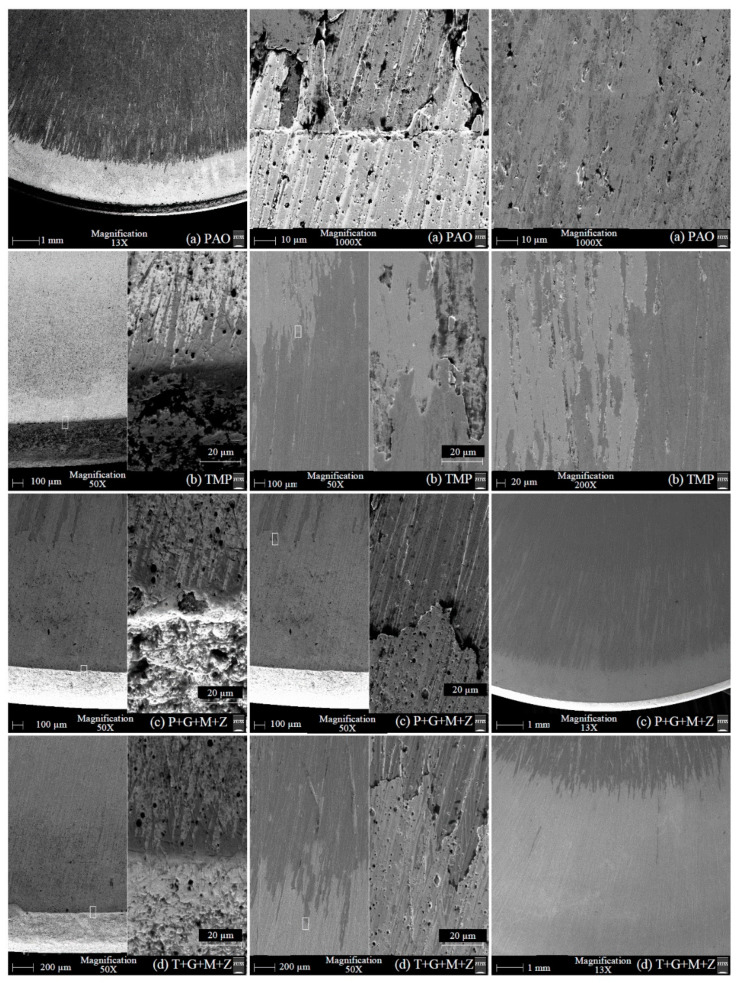
SEM micrographs of a-C:H:W-coated tappets after testing under various conditions in combination with a-C:H:W-coated camlobes in the presence of PAO-based and TMP-based lubricants.

SEM micrographs of a-C:H:W coated tappets are presented in Figure 19. Moreover, some elements deposited on DLC-coated tappets were also noted after the engine testing, of which the atomic percentages are given in Table 6. When PAO was used as the lubricant, it was observed that most of the top carbon layer deteriorated and resulted in an exposed CrN interlayer and iron substrate. In Figure 19b, three distinct layers, comprising the top a-C:H:W layer (dark gray layer at top left and top right corners), the CrN interlayer (light gray layer at the top center), and the iron substrate (white layer at the bottom), can be clearly seen. As a result, the a-C:H:W-a-C:H:W interface was changed to the CrN-CrN and/or U–U interface. The presence of chromium, nitrogen, and ferrous in higher concentrations and a reduction in the percentage of carbon and tungsten compared to the as-deposited a-C:H:W coating in EDS analysis also confirmed severe deterioration. It was observed that the wear resistance of the a-C:H:W coating improved significantly when using additive-free TMP as a lubricant instead of PAO. The removal of the top DLC layer was also seen in the presence of TMP, resulting in an exposed CrN interlayer and ferrous substrate, but not to the extent of PAO. In addition to delamination, pitting and abrasive wear was also seen as a predominant wear mechanism when additive-free lubricants were utilized. Additive-containing lubricants resulted in improvements in the structural integrity of the DLC coating; thus, its delamination was avoided, especially at the outer boundary of the tappet. Furthermore, the nose wear of the DLC-coated camlobe was reduced considerably when multi-additive-containing lubricants were used. This enhanced wear behavior may occur because the graphitization phenomenon becomes less likely in the presence of additives and because an additive-derived tribofilm is formed. EDS analysis also confirmed the development of an additive-derived tribofilm, and this film was composed of molybdenum, sulfur, phosphorus, and zinc.

### 3.8. Surface Roughness Analysis of the a-C:H:W-a-C:H:W Interface

**Figure 20:**
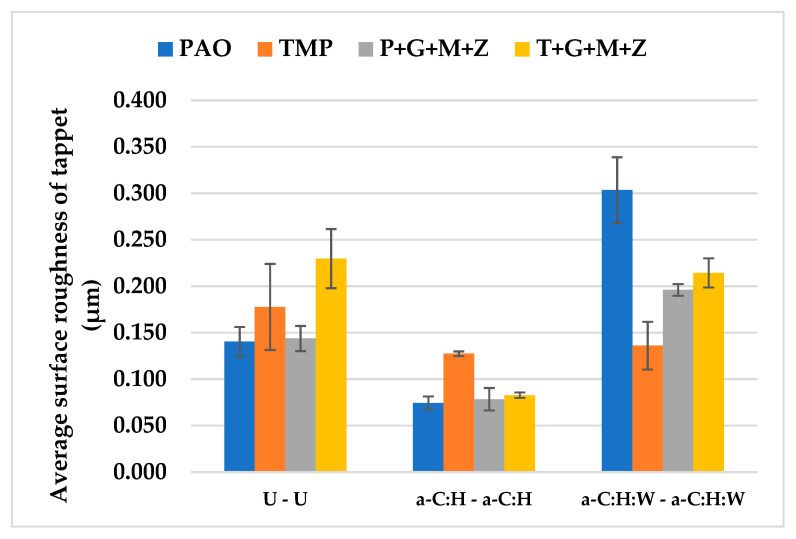
Average surface roughness of uncoated and DLC-coated tappets after cylinder head testing under various conditions in combination with uncoated and DLC-coated camlobes in the presence of PAO-based and TMP-based lubricants.

The R_a_ values of uncoated tappets against uncoated camlobes and of a-C:H:W-coated tappets against a-C:H:W-coated camlobes using two different base oils and two different formulated lubricants were noted and are represented in Figure 20.

Contrary to the U-U and a-C:H-a-C:H interfaces, more deterioration in the a-C:H:W coating was witnessed in the presence of PAO compared to TMP. This response may be due to the fact that the DLC coating was completely delaminated in the presence of PAO, resulting in an exposed ferrous substrate, whereas delamination of the coating was avoided especially at the outer boundary of the tappet when the lubricant was changed to TMP. The wear resistance of the coating was improved to some extent in the presence of the formulated version of PAO, but the surface roughness value was still higher than that of additive-free TMP. On the contrary, a decrease in the surface roughness value was not seen when the lubricant was changed from additive-free TMP to its additive-containing version (T+G+M+Z). Rather, the surface roughness value of a-C:H:W-coated tappets increased by 50% when the lubricant was changed from TMP to T+G+M+Z. This difference in the surface protection capability of the same additives when mixed with different base oils can be attributed to variations in the rotational speeds of the tappets during cylinder head testing. It was observed that the tappets were rotating at higher speeds in the presence of P+G+M+Z compared to T+G+M+Z, irrespective of the lubricant temperature and camshaft speed.

The results and tests were used to assess the friction and wear performance of chemically-modified palm oil (TMP). For the effective use of palm oil, sustainability business strategies require integration into the business models of palm oil companies [39]. Palm oil faces various issues in the global market. Malaysia has launched the Malaysian Sustainable Palm Oil (MSPO) certification as the national scheme to systematically promote and certify the palm oil industry in Malaysia as a step towards sustainable production, as well to address some of the concerns of the global market, such as the requirements of importing countries [40].

## 4. Conclusions

The purpose of this study was multifold. The study was primarily performed to investigate the potential of bio-based lubricants as potential replacements for conventional engine oils to develop environment-friendly sustainable solutions and tribological performance enhancements of the current mating engine components by experimentally simulating real engine operating conditions. This study covered the tribological analysis of TMP in combination with conventional additives and two different types of DLC coatings, while comparing both base oils and the formulated lubricants of PAO for the tappet/cam interface of the direct acting valve train assembly of a diesel engine. Some of the important conclusions of this experimental study are given below:

It was observed that friction torque between the camlobe and tappet decreased with a decrease in the lubrication temperature and an increase in the camshaft speed. During cylinder head testing, the lowest values of friction torque were observed for the a-C:H-coated tappet and the a-C:H- coated camlobe interface, whereas the maximum friction was seen with the U-U interface. Among the tested lubricants, P+G+M+Z was the most effective in mitigating the friction between the cam and tappet interface, whereas PAO proved to be the least effective.

Among the different interfaces, the highest wear performance was observed for the a-C:H-a-C:H interface. Among the tested lubricants, T+G+M+Z was the most effective in protecting the tappets from surface deterioration and excessive wear, whereas TMP resulted in aggravated wear. The maximum values of nose wear of the camlobe were observed when PAO was used as the lubricant, whereas P+G+M+Z resulted in minimum nose wear values.

In optical images of uncoated and DLC-coated tappets after cylinder head testing, it was observed that the surface deteriorated greatly in the uncoated tappets, especially when additive-free lubricants were used. On the other hand, it was observed that the a-C:H coating maintained its structural integrity throughout the test and delamination was not observed, irrespective of lubricant type. Similar wear behavior was also seen in camlobes. However, some of the coating was also removed from the right edge of the nose of a-C:H-coated camlobes as well. In the case of camlobes, the coating was only delaminated from the nose area and cam flanks, whereas it remained intact on the base circle.

Based on these conclusions, it can be summarized that a-C:H and TMP have the potential to be used as surface-protective coatings and lubricant base oils, respectively, for the tappet/cam interface of a direct acting valve train assembly. However, there is a need to evaluate other engine assemblies in combination with the formulated TMP to further validate its potential. Moreover, conventional lubricant additives are tribologically compatible with TMP and the a-C:H coating to some extent, but there is a need to manufacture dedicated additives for bio-based oils.

## Figures and Tables

**Table 1:** Physicochemical properties and formulation details of formulated and additive-free lubricants.

Lubricants	Formulation Details					Physicochemical Properties		
	TMP (wt.%)	PAO (wt.%)	GMO (wt.%)	MoDTC (wt.%)	ZDDP (wt.%)	Kinematic Viscosity at 100 °C (cSt)	Viscosity Index	Density (g/cm^3^)
TMP	100	-	-	-	-	9.33	194.50	0.92
PAO	-	100	-	-	-	9.85	135.40	0.84
TMP+GMO+MoDTC+ZDDP (T+G+M+Z)	97	-	1	1	1	9.84	193.24	0.92
PAO+GMO+MoDTC+ZDDP (P+G+M+Z)	-	97	1	1	1	9.98	135.34	0.838

**Table 2:** Properties of a-C:H coatings.

Properties	a-C:H	a-C:H:W
Deposition technique	PACVD combined with ion sputtering	PVD combined with ion sputtering
Interlayer	CrN	CrN
Thickness	2–3 µm	2–3 µm
Average surface roughness	0.02–0.03 µm	0.02–0.03 µm
Hardness	15–25 GPa	12–15 GPa
Maximum service temperature	300 °C	300 °C
Color	Black	Anthracite

**Table 3:** OM646LA diesel engine specifications.

Manufacturer	Daimler
Displacement	2148 cubic centimeter (cc)
Fuel	Common rail diesel direct injection
Induction	Turbocharged
Number of cylinders	4′
Cylinder configuration	In-line
Emission compliance level	Euro IV
Exhaust post-treatment method	Oxidation catalyst converter (Oxy-Cat)/diesel particulate filter (DPF)

**Table 4:** Test matrix for cylinder head testing.

Test No.	Lubricant	Tribopair	Lubricant Temperature	Camshaft Speeds
New uncoated camlobe/uncoated tappet pair and fresh additive-free PAO				
**1.**	PAO	Uncoated cam/uncoated tappet	40 °C	400 RPM, 800 RPM, and 1200 RPM
**2.**	PAO	Uncoated cam/uncoated tappet	90 °C	400 RPM, 800 RPM, and 1200 RPM
New a-C:H-coated camlobe/a-C:H-coated tappet pair				
**3.**	PAO	a-C:H-coated camlobe/a-C:H-coated tappet	40 °C	400 RPM, 800 RPM, and 1200 RPM
**4.**	PAO	a-C:H-coated camlobe/a-C:H-coated tappet	90 °C	400 RPM, 800 RPM, and 1200 RPM
New a-C:H:W-coated camlobe/a-C:H:W-coated tappet pair				
**5.**	PAO	a-C:H:W-coated camlobe/a-C:H:W-coated tappet	40 °C	400 RPM, 800 RPM, and 1200 RPM
**6.**	PAO	a-C:H:W-coated camlobe/a-C:H:W-coated tappet	90 °C	400 RPM, 800 RPM, and 1200 RPM
New uncoated camlobe/uncoated tappet pair and fresh additive-free TMP				
**7.**	TMP	Uncoated cam/uncoated tappet	40 °C	400 RPM, 800 RPM, and 1200 RPM
**8.**	TMP	Uncoated cam/uncoated tappet	90 °C	400 RPM, 800 RPM, and 1200 RPM
New a-C:H-coated camlobe/a-C:H-coated tappet pair				
**9.**	TMP	a-C:H-coated camlobe/a-C:H-coated tappet	40 °C	400 RPM, 800 RPM, and 1200 RPM
**10.**	TMP	a-C:H-coated camlobe/a-C:H-coated tappet	90 °C	400 RPM, 800 RPM, and 1200 RPM
New a-C:H:W-coated camlobe/a-C:H:W-coated tappet pair				
**11.**	TMP	a-C:H:W-coated camlobe/a-C:H:W-coated tappet	40 °C	400 RPM, 800 RPM, and 1200 RPM
**12.**	TMP	a-C:H:W-coated camlobe/a-C:H:W-coated tappet	90 °C	400 RPM, 800 RPM, and 1200 RPM
New uncoated camlobe/uncoated tappet pair and formulated TMP (T+G+M+Z)				
**13.**	T+G+M+Z	Uncoated cam/uncoated tappet	40 °C	400 RPM, 800 RPM, and 1200 RPM
**14.**	T+G+M+Z	Uncoated cam/uncoated tappet	90 °C	400 RPM, 800 RPM, and 1200 RPM
New a-C:H-coated camlobe/a-C:H-coated tappet pair				
**15.**	T+G+M+Z	a-C:H-coated camlobe/a-C:H-coated tappet	40 °C	400 RPM, 800 RPM, and 1200 RPM
**16.**	T+G+M+Z	a-C:H-coated camlobe/a-C:H-coated tappet	90 °C	400 RPM, 800 RPM, and 1200 RPM
New a-C:H:W-coated camlobe/a-C:H:W-coated tappet pair				
**17.**	T+G+M+Z	a-C:H:W-coated camlobe/a-C:H:W-coated tappet	40 °C	400 RPM, 800 RPM, and 1200 RPM
**18.**	T+G+M+Z	a-C:H:W-coated camlobe/a-C:H:W-coated tappet	90 °C	400 RPM, 800 RPM, and 1200 RPM
New uncoated camlobe/uncoated tappet pair and fresh formulated PAO (P+G+M+Z)				
**19.**	P+G+M+Z	Uncoated cam/uncoated tappet	40 °C	400 RPM, 800 RPM, and 1200 RPM
**20.**	P+G+M+Z	Uncoated cam/uncoated tappet	90 °C	400 RPM, 800 RPM, and 1200 RPM
New a-C:H-coated camlobe/a-C:H-coated tappet pair				
**21.**	P+G+M+Z	a-C:H-coated camlobe/a-C:H-coated tappet	40 °C	400 RPM, 800 RPM, and 1200 RPM
**22.**	P+G+M+Z	a-C:H-coated camlobe/a-C:H-coated tappet	90 °C	400 RPM, 800 RPM, and 1200 RPM
New a-C:H:W-coated camlobe/a-C:H:W-coated tappet pair				
**23.**	P+G+M+Z	a-C:H:W-coated camlobe/a-C:H:W-coated tappet	40 °C	400 RPM, 800 RPM, and 1200 RPM
**24.**	P+G+M+Z	a-C:H:W-coated camlobe/a-C:H:W-coated tappet	90 °C	400 RPM, 800 RPM, and 1200 RPM

**Table 5:** Elemental atomic percentage found on a-C:H-coated tappets post-cylinder head testing under various conditions in combination with a-C:H-coated camlobes with TMP-based and PAO-based lubricants.

Lubricants	Elements									
	C	Cr	N	P	S	Mo	Zn	Fe	O	Ar
PAO	97.07	-	-	-	-	-	-	-	2.52	0.41
TMP	64.52	7.61	3.41	-	-	-	-	11.72	12.74	-
P+G+M+Z	94.41	0.67	-	0.12	0.32	1.66	1.33	-	1.49	-
T+G+M+Z	79.33	7.94	2.98	0.12	0.25	1.03	0.37	-	7.98	-

**Table 6:** Elemental atomic percentage found on a-C:H:W-coated tappets after cylinder head testing under various conditions in combination with a-C:H:W-coated camlobes in the presence of PAO-based and TMP-based lubricants.

Lubricants	Elements						
	C	W	Cr	N	Fe	O	Ni
PAO	35.3	7.3	4.8	6.0	32.5	10.7	3.4
TMP	60.1	14.9	6.2	2.4	2.7	9.0	4.6
P+G+M+Z	39.6	11.5	10.1	9.2	21.9	3.4	4.2
T+G+M+Z	11.9	0.4	11.0	10.7	57.3	8.7	-

## Data Availability

Not applicable.
